# Fluoroscopically guided jejunal tube placement via percutaneous gastrostomy in children: technical success, safety, and procedural parameters

**DOI:** 10.1007/s00247-026-06572-8

**Published:** 2026-03-12

**Authors:** Michael Esser, Jakob Spogis, Johannes Hilberath, Jürgen F. Schäfer, Ilias Tsiflikas

**Affiliations:** 1 Department of Diagnostic and Interventional Radiology, Pediatric Radiology, University Hospital Tübingen, Hoppe-Seyler-Str. 3, Tübingen, 72076 Germany; 2 Pediatric Gastroenterology and Hepatology, Department of Haematology and Oncology, University Children’s Hospital Tübingen, Tübingen, Germany

**Keywords:** Enteral nutrition, Gastric feeding tubes, Gastrostomy, Interventional radiography, Intestinal diseases, Pediatrics

## Abstract

**Background:**

Fluoroscopically guided jejunal tube placement via percutaneous endoscopic gastrostomy (PEG-J) provides minimally invasive post-pyloric access in children. Limited data exist regarding routine application and procedural risks.

**Objective:**

To evaluate the safety and technical success of PEG-J in pediatric patients, performed without general anesthesia or sedation.

**Materials and methods:**

All pediatric cases of fluoroscopically guided PEG-J procedures performed between 2011 and 2025 were included. Fluoroscopic images were reviewed to determine the final position of the tube tip. Technical success, complications, anatomical variants, and tube patency were assessed. Fluoroscopy time and dose area product (DAP) were documented.

**Results:**

A total of 126 PEG-J procedures in 60 children (36 males) were analyzed. The technical success rate was 85% (107/126) with final tube tip placement in the jejunum in 88 cases (82%) and in the duodenum in 19 cases (18%). Nineteen procedures (15%) were unsuccessful, including six with documented anatomical causes (steep vertical duodenal entry, *n*=2; malrotation, hiatus hernia, hooked stomach in superior mesenteric artery syndrome, steep take-off of the jejunum with kinking of the tube at the ligament of Treitz, *n*=1 each) and 13 without documented reasons. The median fluoroscopy time was 5 min 24 s (range, 2 s–37 min), at a frame rate of 0.5 frames per second. The median DAP was 6.1 cGy·cm^2^ (range, 0.08–343 cGy·cm^2^).

**Conclusion:**

Fluoroscopically guided PEG-J placement is a safe and effective procedure in pediatric patients, with high technical success and low radiation exposure.

**Graphical Abstract:**

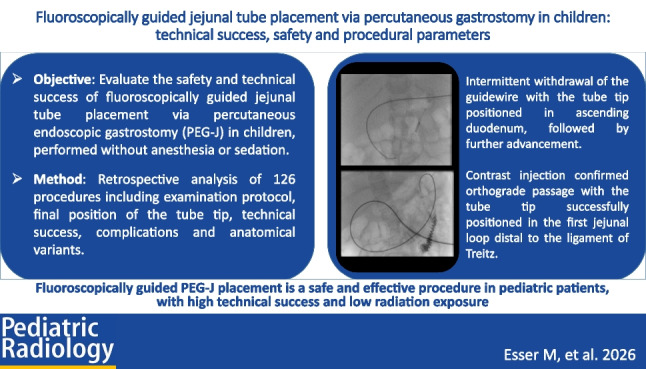

## Introduction

Enteral nutrition is the preferred method of nutritional support in pediatric patients with insufficient oral intake, particularly in the context of complex medical conditions or critical illness. Gastric tube feeding is commonly used because of its simplicity and ability to replicate physiological feeding patterns. However, in a subset of children - particularly those with severe gastroesophageal reflux disease, lack of gag reflex, or gastric dysmotility - gastric feeding is poorly tolerated or contraindicated. In such cases, jejunal feeding provides a valuable alternative, by enabling post-pyloric nutrient delivery and reducing reflux-related complications and aspiration risk [1, 2].

In acute care settings, particularly in pediatric intensive care units, jejunal feeding is often initiated using nasojejunal or transpyloric tubes to provide short-term post-pyloric nutrition [3, 4]. These tubes are intended for temporary use and are prone to dislodgement and limited durability, making them unsuitable for long-term enteral support in chronically ill children.

According to the 2019 position paper of the European Society for Paediatric Gastroenterology, Hepatology and Nutrition, long-term jejunal feeding in children can be achieved using endoscopic, radiological, or surgical techniques, most commonly via a gastrostomy or gastrojejunostomy [5, 6]. However, these techniques pose specific challenges in pediatrics, where patient size, anatomy, and tolerance to procedural stress vary widely. In pediatric practice, endoscopic approaches often require general anesthesia to ensure immobility and airway protection, particularly in younger or neurologically impaired children [7]. Repeated or prolonged exposure to anesthesia in early childhood has raised concerns regarding potential neurodevelopmental effects, prompting the need for alternative strategies in high-risk or vulnerable pediatric populations [8].

More recently, ultrasound-guided techniques for post-pyloric tube placement have been described, primarily for temporary nasoenteric feeding in intensive care settings. However, these approaches are highly operator-dependent and have limited applicability for long-term jejunal feeding or tube advancement via a pre-existing gastrostomy [3, 9]. In this context, fluoroscopically guided placement of jejunal extension tubes via pre-existing percutaneous endoscopic gastrostomy (PEG), commonly referred to as PEG-J, represents a pragmatic approach for long-term jejunal feeding. This technique allows controlled post-pyloric advancement under real-time visualization, often beyond the ligament of Treitz, while avoiding the need for general anesthesia [10, 11]. While fluoroscopic PEG-J placement is well established in adults [12], pediatric data remain scarce. To date, only a single small pediatric case series has specifically described fluoroscopic advancement of a jejunal tube through an existing gastrostomy [13]. Consequently, systematic pediatric data on procedural parameters - such as fluoroscopy time, radiation exposure, and the impact of anatomical variants - are largely lacking, particularly across a broad age range.

Therefore, this study aimed to evaluate the technical success, safety profile, and procedural characteristics of fluoroscopically placed post-pyloric tubes via pre-existing PEG in a pediatric cohort.

## Materials and methods

This retrospective single-center study was approved by the local ethics committee, which waived the requirement for informed consent. All procedures were performed in accordance with the Guidelines for Good Clinical Practice and the ethical standards of the 1964 Declaration of Helsinki and its later amendments, or comparable ethical standards.

### Patient selection

A retrospective patient cohort was selected from our radiology information system between January 2011 and January 2025. The study included all pediatric patients (under 18 years of age) who underwent fluoroscopically guided post-pyloric tube placement via pre-existing gastrostomy at our institution. Search terms related to the procedure (“PEG-J,” “Percutaneous Endoscopic Jejunostomy,” “PEJ,” “jejunal tube,” and “duodenal tube”) were used to identify examinations from the electronic medical record system. Procedures conducted outside the fluoroscopy unit were excluded. Procedures performed under general anesthesia or sedation were excluded from the analysis. During the study period, four patients underwent jejunal tube placement with sedation and were therefore not included. The excluded procedures performed under sedation were related to pronounced agitation or excessive hyperactivity; therefore, sedation may be considered in such cases, although an initial attempt without sedation is generally recommended whenever feasible. The main clinical indication for jejunal tube placement was gastric feeding intolerance due to gastric dysmotility and/or severe gastroesophageal reflux with or without aspiration risk; in one patient, duodenal decompression was an additional indication (as outlined in the “Results”). Demographic characteristics are shown in Table 1.

**Table 1:** Patient demographics and procedural characteristics

Total number of patients/procedures	60/126
Sex, *n* (%)	Female, 24 (40%) Male, 36 (60%)
Age, years, median (range)	6.9 (0.25–17.9)
Age groups, *n* (%)	
• Group 1, ≤1 year (infants)	22 (18%)
• Group 2, 2–5 years	36 (29%)
• Group 3, 6–10 years	32 (25%)
• Group 4, 11–15 years	28 (22%)
• Group 5, 16–17 years	8 (6%)
Procedures per patient, n, mean (range)	2 (1–17)
Freka® Intestinal Tube/G-Jet® Button, *n* (%)	117 (93%)/9 (7%)
First tube insertion/replacement/exchange, *n* (%)	36 (29%)/31 (24%)/59 (47%)
Technical success rate, *n* (%)	107/126 (85%)
Full success/partial success, *n* (% of all successful)	88 (82%)/5 (18%)
Failed placements, *n* (%)	19 (15%)
Anatomic anomalies (failed), *n* (% of all failed)	6/19 (32%)
Fluoroscopy time, min, median; IQR; range	5.4; 8.2; 0.03–37
DAP, cGy·cm^2^, median; IQR; range	6.1; 8.8; 0.08–343

*n* number, *DAP* dose area product, *IQR* interquartile range

To allow for age-related analysis and dose comparison, patients were stratified into five age groups according to anthropometric and radiological standards, based on growth data [14] and German diagnostic reference levels [15]:

- Group 1, ≤1 year (infants)
- Group 2, 2–5 years
- Group 3, 6–10 years
- Group 4, 11–15 years
- Group 5, 16–17 years

### Procedure

All procedures were performed by or under the supervision of board-certified pediatric radiologists: I.T. (14 years of experience; 86 procedures within the observation time), J.F.S. (14 years; 16 procedures), and M.E. (10 years; 24 procedures). Oral informed consent from the parents was obtained for this procedure. No dedicated pre-procedural imaging was routinely performed prior to initial PEG-J placement. Images from prior placements and upper gastrointestinal contrast studies were reviewed when present, but were not required for proceeding with the fluoroscopic procedure. All tubes were placed under real-time fluoroscopy guidance. The radiation field was confined precisely to the region of interest using cones aligned with a light-beam collimator.

All procedures were performed using one of the following C-arm-equipped flat detector fluoroscopy units with automatic exposure control and an integral DAP meter:

- Philips MultiDiagnost Eleva FD 2.0 (Philips Medical Systems, Netherlands)
- Siemens Artis zee multipurpose fluoroscopy system (Siemens Healthineers, Erlangen, Germany)

Two types of jejunal tubes were used:

- Freka® Intestinal Tube FR9 ENFit (Fresenius Kabi AB, Bad Homburg, Germany), polyurethane tube (120 cm, CH/FR 9)
- G-Jet® Button (Applied Medical Technology, Inc., Brecksville, OH)

All Freka® Intestinal Tubes were placed via a pre-existing PEG tube (CH15). Initial advancement was performed over a pre-inserted, hydrophilic, Teflon-coated guidewire (supplied by the manufacturer as part of the Freka® Intestinal Tube Set) with a straight, flexible tip (1.2 mm), used to straighten the distal tube tip and improve navigability. Patients were positioned supine; right lateral decubitus positioning was selectively used to facilitate transpyloric passage. Gastric air insufflation (to improve visualization of gastric contours and orientation toward the pylorus), particularly in fasting patients or when the stomach was decompressed via PEG drainage, and small volumes of saline or sterile water (to promote gastric emptying and stimulate peristalsis when spontaneous advancement of the tube did not occur) were selectively administered through the jejunal limb; the gastrostomy tract itself served only as an access route. After reaching the duodenum, the guidewire was partially withdrawn (approximately 1 cm) in the horizontal or, at the latest, in the ascending portion of the duodenum to prevent premature loop formation of the tube tip and to facilitate passage around the ligament of Treitz while maintaining adequate steerability.

G-Jet® Buttons were placed either by primary wire-guided insertion through the existing gastrostomy tract or (after removal of the gastric tube), in cases of replacement, using the Seldinger technique over a separate guidewire (Merit Laureate® Hydrophilic Guide Wire Stiff Shaft, Angled Stiff, diameter 0.035 in. (0.89 mm), length 180 cm, flexible tip length 3 cm; Merit Medical, South Jordan, UT). Following balloon deflation and removal of the previous device, the new button was advanced under fluoroscopic control with the jejunal limb beyond the ligament of Treitz. The gastric retention balloon was inflated according to the manufacturer’s instructions.

Regardless of the tube type, final tube position was confirmed by contrast injection (Ultravist®, diluted 2:1 with saline) demonstrating orthograde intrajejunal flow. In cases where pyloric passage was difficult or air insufflation did not sufficiently delineate the gastric outlet, early contrast injection was used selectively. Afterwards, the tube was flushed with water or saline. In cases involving anatomical variants, no alternative catheter types or guidewires were used. Tube looping was managed by partial withdrawal and guidewire advancement to restore a straight trajectory; reinsertion was performed if displacement into the stomach occurred. Procedures were aborted when post-pyloric advancement was not technically feasible due to anatomical or tube-related factors, or when safe continuation was precluded by reduced cooperation; fluoroscopy time served as an orienting parameter but was not a fixed stopping criterion. Final tube position was documented. The closure system was secured according to the manufacturer’s instructions.

Fluoroscopy time and dose area product (DAP) recorded from the integral DAP meter were extracted directly from the modality protocol.

### Data collection

Patient demographics, including age at the time of procedure and sex, procedural data, and outcome measures, were collected from electronic medical records and radiology reports. Procedures were categorized as first-time (new) insertion, replacement (unplanned exchange due to tube dislocation or loss of patency), or exchange (scheduled tube change at an individually defined interval); for subgroup analysis, replacement and exchange procedures were combined and compared with first-time insertions (Table 2).

**Table 2:** Fluoroscopy time and dose area product (*DAP*) by age group and procedure type

Age group	Fluoroscopy time, min, median; IQR^a^; range			DAP, cGy·cm^2^, median; IQR^a^; range		
	All procedures	First insertion	Replacement	All procedures	First insertion	Replacement
≤1 year	6.2; 7.8; 0.6–21.5.6.5	6.5; 7.6; 3.6–19.5.6.5	3.2; 8.8; 0.6–21.5.6.5	3.8; 4.2; 0.2–30.2	5.8; 3.3; 1.5–8.9.5.9	2.4; 3.4; 0.2–30.2
2–5 years	5.8; 8.2; 0.1–20.8.1.8	8.2; 12.8; 0.1–20.8.1.8	5.6; 7.6; 0.1–15.5.1.5	5.4; 8.7; 0.1–23.1.1.1	5.5; 9.1; 0.1–23.1.1.1	5.3: 7.4; 1.1–21.8.1.8
6–10 years	4.5; 7.9; 0.03–22.2.03.2	9.6; 3.6; 6.5–11.7.5.7	3.7; 7.4; 0.03–22.2.03.2	5.8; 11.4; 0.1–49.9.1.9	13.2; 9.9; 7.5–22.7.5.7	3.6; 8.6; 0.1–49.9.1.9
11–15 years	4.9; 11.7; 0.1–27.1	14.7; -; 7.3–19.3	4.6; 10.7; 0.1–27.1	7.5; 19.9; 0.7–198.4.7.4	17.7; -; 9.8–63.8.8.8	6.9; 20; 0.7–198.4.7.4
16–17 years	4.8; 25.5; 0.3–37.3	31.2; -; 12.2–37.2	1.8; 4.3; 0.3–5.3.3.3	11.6; 63.3; 1.9–343.9	80.8; -; 22.8–343.8	4.4; 9.4; 1.9–14.3.9.3

*IQR* interquartile range

^a^IQR is not shown in subgroups with very small sample sizes, where quartiles could not be meaningfully determined

Technical success of tube placement was defined as the post-pyloric positioning of the tube tip, with a distinction made between placement within the duodenum (partial success) and optimal target position beyond the ligament of Treitz (full success). The final position of the tube tip was determined by retrospectively reviewing the fluoroscopic images from each procedure. All examinations were reviewed in a consensus reading by two board-certified pediatric radiologists with 14 years (I.T.) and 10 years of experience (M.E.) on a dedicated workstation. Failure of tube placement was defined as any tube that did not exit the stomach or any aborted intervention. Reasons for unsuccessful placement were documented, if applicable. The patients’ records were reviewed for any complications after tube placement related to the intervention, mode and timing of previous jejunal tube placement (endoscopic or fluoroscopic) and subsequent tube placement strategy in unsuccessful cases. Where available, endoscopy reports were analyzed for relevant anatomical abnormalities (e.g. postsurgical changes, duodenal variants, malrotation).

### Statistical analysis

Statistical analysis was performed using IBM SPSS Statistics (Version 28.0 for Windows, IBM Corp., Armonk, NY). The Kolmogorov–Smirnov test was applied to assess the normality of continuous variables. Data with normal distribution are presented as mean±standard deviation (SD). Non-normally distributed variables are reported as median with interquartile range (IQR). In subgroups with very small sample sizes or identical values, the interquartile range could not be meaningfully determined and is therefore not shown. Descriptive statistics were used for categorical variables. Group comparisons (e.g., age groups, procedure success) were performed using appropriate parametric or non-parametric tests as indicated. A *P*-value less than 0.05 was considered statistically significant.

Subgroup analyses were predefined to compare procedural parameters between successful and unsuccessful placements. In addition, a subgroup of cases with prolonged fluoroscopy time (>15 min) was analyzed separately. Fluoroscopy time and DAP were analyzed stratified by age group, by procedure type (new insertion vs. replacement) and by success (full success, partial success, failed).

Exploratory comparisons between examiners with respect to fluoroscopy time and DAP were performed using the Kruskal–Wallis test.

## Results

A total of 126 fluoroscopically guided PEG-J placements were performed in 60 pediatric patients (36 males) between 2011 and 2025. The median age at the time of intervention was 6.7 years (IQR, 9.1 years; range, 3 months to 17 years), with a mean of 2 procedures per patient (SD, 2.8; range, 1–17). Detailed patient demographics and procedural characteristics are summarized in Table 1.

### Technical success

**Fig. 1:**
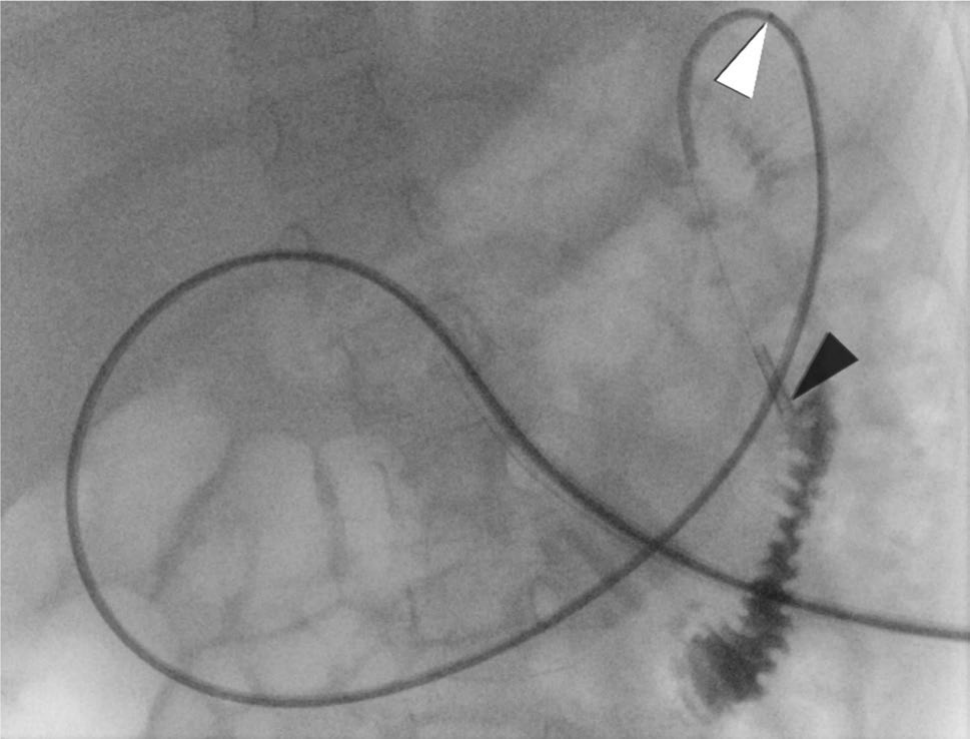
6-year-old girl with metachromatic leukodystrophy and feeding difficulties undergoing a scheduled tube exchange, demonstrating successful jejunal tube placement. Anteroposterior fluoroscopic image of the upper abdomen demonstrating fluoroscopy-guided jejunal tube placement with a dose area product (DAP) of 1.3 cGy·cm^2^ and a fluoroscopy time of 1.2 min. The tube tip (*black arrowhead*) is positioned within the first jejunal loop, with the guidewire partially withdrawn to the level of the ligament of Treitz (*white arrowhead*). Contrast injection confirms orthograde contrast passage within the jejunum

The overall technical success rate of post-pyloric tube placement was 85% (107/126; Tables 1 and 3). Among successful procedures, the final tube tip position was located in the jejunum in 88 cases (82%; Fig. 1) classified as full success. In 18 cases (17%), the tube tip was positioned in the ascending duodenum. In 14 of these interventions (one patient), duodenal position was intentionally selected due to prior surgical closure at the level of the ligament of Treitz, with PEG-J serving as a decompression tube. These procedures were consistently classified as partial technical success according to the predefined anatomical criteria. In one case (1%) with known intestinal malrotation, elastic resistance and reduced navigability prevented further distal advancement; the tube was therefore left in the descending duodenum and classified as partial success. Finally, 19 placements (18% among successful procedures) were classified as partial success. A further 19 procedures (15% of all procedures) were unsuccessful in achieving post-pyloric positioning. Patient characteristics and procedural parameters for the respective subgroups are presented in Table 2.

**Table 3:** Patients characteristics and procedural parameters by full success, partial success, and failed placement

	Full success	Partial success	Failed placement
Number of procedures	88	19	19
Sex, *n*	Female, 39 Male, 49	Female, 3 Male, 16	Female, 4 Male, 15
Age, years, median (range)	5.8 (0.5–17.7.5.7)	10 (3–14.4.4)	8.3 (0.25–17.9.25.9)
First time/replacement	27/61	2/17	7/12
Fluoroscopy time, min, median; IQR; range	5.6; 7.6; 0.03–27.03	2.9; 4.5; 0.1–21.8.1.8	10.1; 11.5; 0.1–37.1
DAP, cGy·cm^2^, median; IQR; range	5.8; 7.8; 0.1–198.1	3.7; 5; 0.3–14.3	11.9; 23.6; 0.2–343.2

*DAP* dose area product,* IQR* interquartile range

**Fig. 2:**
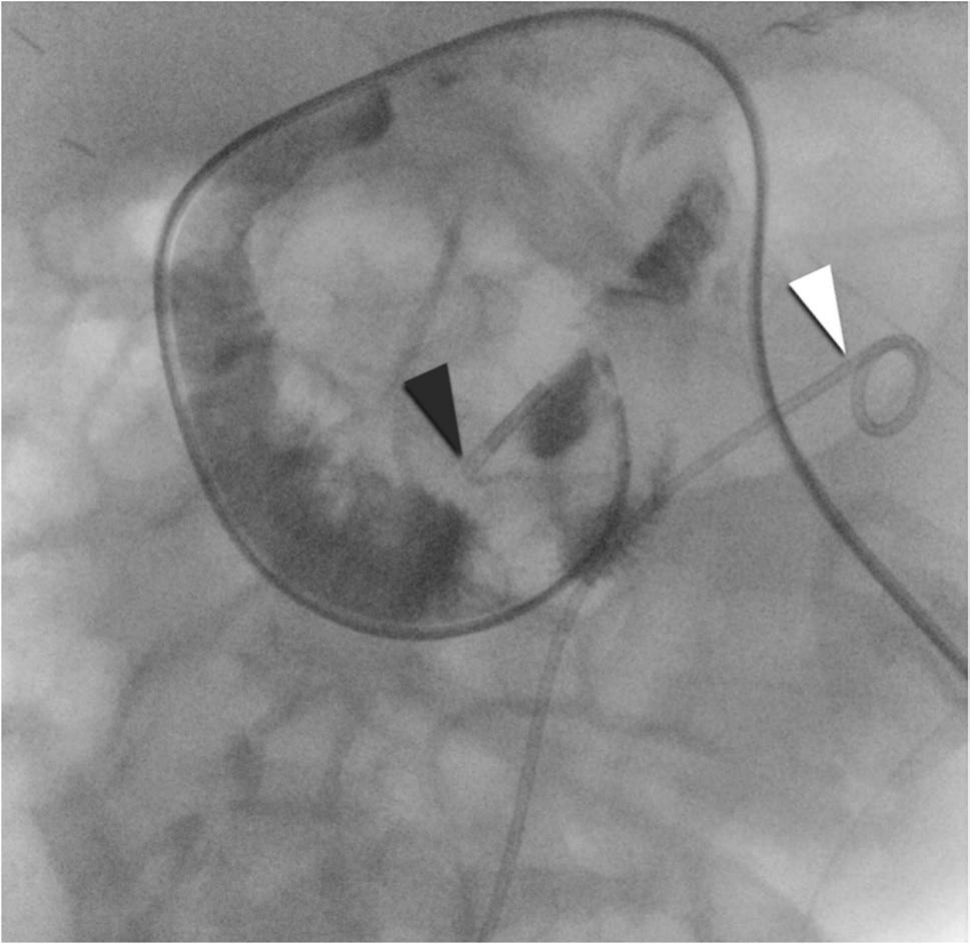
12-month-old boy with Perlman syndrome and severe gastroparesis, resulting in failure of adequate enteral nutrition via nasogastric tube. Anteroposterior fluoroscopic image of the upper abdomen obtained during an attempted fluoroscopy-guided jejunal tube placement, resulting in a dose area product (DAP) of 30 cGy·cm.^2^ and a fluoroscopy time of 21.5 min. The tube was advanced with a slightly withdrawn guidewire through the contrast-opacified duodenum. At the level of the duodenojejunal junction, the tube tip (*black arrowhead*) repeatedly looped back, and multiple attempts to straighten and advance the tube - including partial withdrawal and re-advancement as well as small-volume contrast injection - were unsuccessful. During these maneuvers, the tube repeatedly recoiled into the stomach, after which the pylorus could no longer be traversed. Endoscopic placement was subsequently performed. A markedly steep origin of the proximal jejunal loop was later confirmed. Note the double-J ureteral stent in the left kidney (*white arrowhead*)

Among the 19 unsuccessful placements, anatomical anomalies were documented in six cases. These included unphysiological anatomy with steep vertical duodenal entry (*n*=2), intestinal malrotation (*n*=1), hiatal hernia (*n*=1), a hooked stomach in the context of superior mesenteric artery syndrome (*n*=1), and a steep origin of the jejunum with tube kinking at the ligament of Treitz (*n*=1; Fig. 2). In 13 additional cases, no specific reason for placement failure was documented.

G-Jet® buttons accounted for a small proportion of procedures (*n*=9), including two first-time insertions and seven scheduled exchanges, including one failed placement (without documented reason) and one partial success (ascending part of duodenum).

### Procedural characteristics

The median fluoroscopy time was 5 min and 24 s (IQR, 8 min 12 s; range, 2 s to 37 min), at a standard frame rate of 0.5 frames per second. The median DAP was 6.1 cGy·cm^2^ (IQR, 8.8 cGy·cm^2^; range, 0.08 cGy·cm^2^ to 343 cGy·cm^2^). Aside from failed placements, no procedure-related complications were reported.

**Fig. 3:**
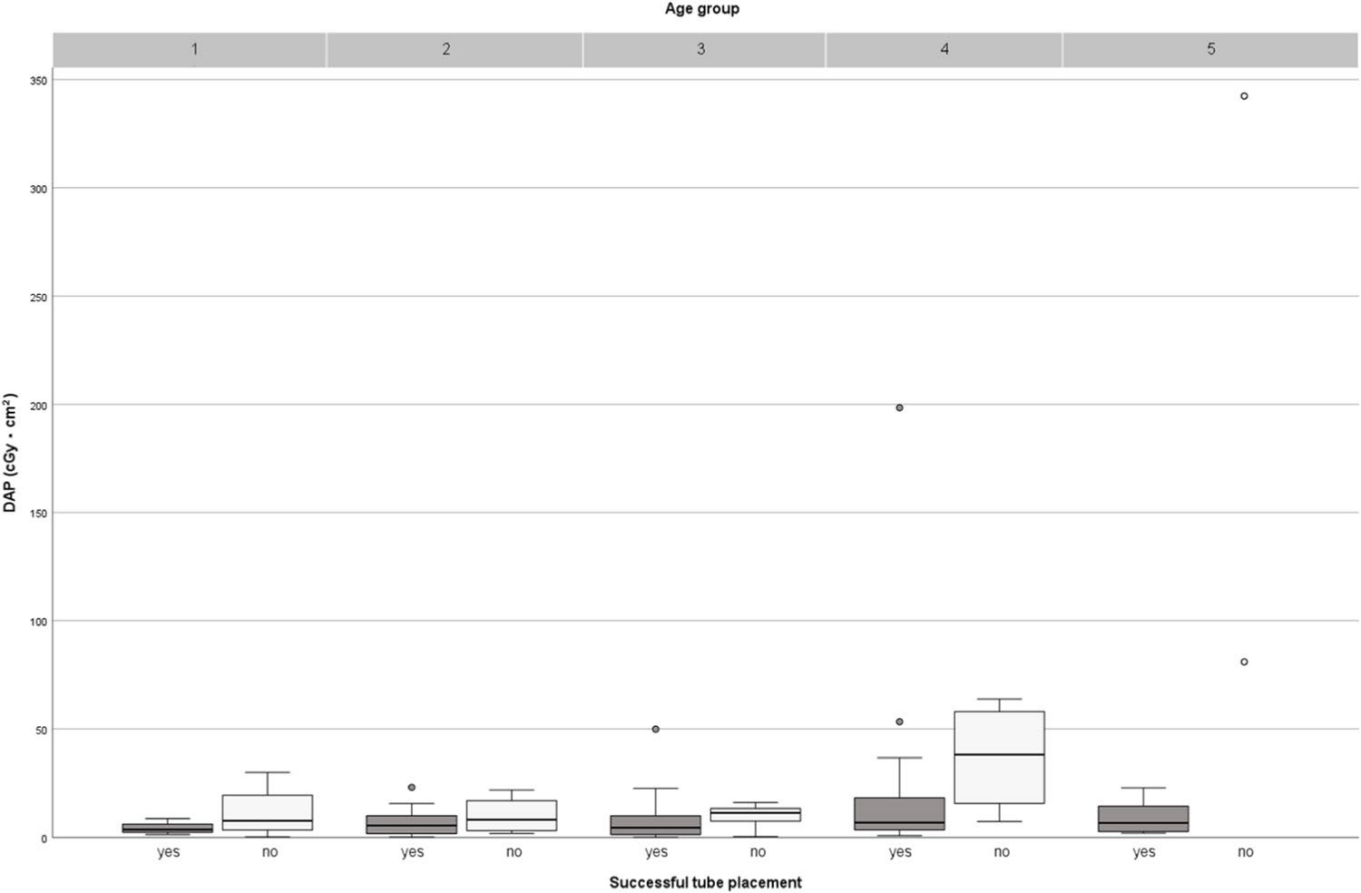
Boxplots of dose area product (DAP, cGy·cm^2^) by age group divided into successful and failed tube placements. The highest DAP values were observed in age groups 4 and 5. Age group 5 included two failed placements, both associated with exceptionally high DAP values (80 cGy·cm^2^ and 343 cGy·cm^2^), identifiable as outliers. Across all age groups, failed placements consistently resulted in higher radiation exposure than successful procedures

**Fig. 4:**
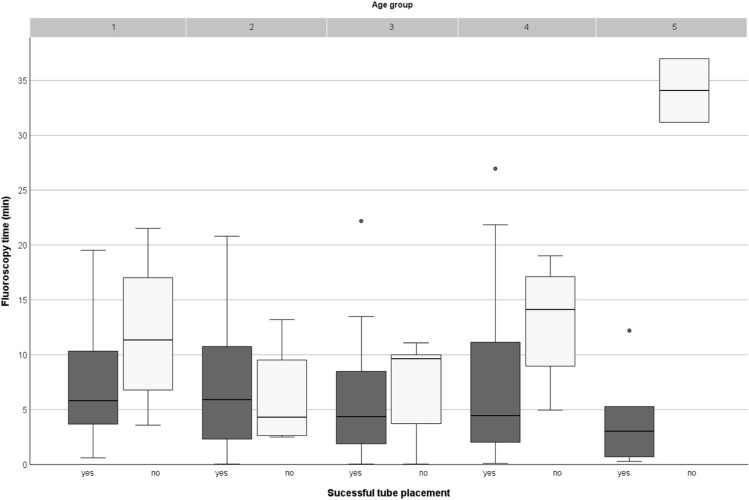
Boxplots of fluoroscopy time (minutes, min) by age group, divided into successful and failed tube placements. Among successful procedures, fluoroscopy time did not demonstrate a consistent increase across age groups; values in older children remained within a comparable range to those in younger patients. Age group 4 showed greater variability due to two successful procedures with prolonged fluoroscopy time (27 min and 21 min). Failed placements were associated with longer fluoroscopy times across most of the age groups, including two outliers in age group 5 exceeding 30 min, which substantially influenced the distribution. *min* minutes

Fluoroscopy parameters were further analyzed by age group. While DAP showed a tendency to increase with patient age, fluoroscopy time demonstrated a less uniform pattern (Table 2). Median fluoroscopy times were relatively similar across the younger age groups (6.2 min in group 1 and 5.8 min in group 2) and were even lower in the following age groups (4.5–4.9 min in groups 3–5). Graphical representations of DAP and fluoroscopy time by age group are shown in Figs. 3 and 4, respectively. Across all age groups, median fluoroscopy time and DAP were, as expected, markedly lower for replacement procedures compared with first-time insertions (Table 2).

Age group 5 (16–17 years) required separate consideration regarding radiation dose and fluoroscopy time. Of the eight cases in this group, two examinations involved unsuccessful tube placements, both of which showed markedly elevated dose values - including the highest recorded DAP values in our cohort (80 cGy·cm^2^ and 343 cGy·cm^2^, respectively) - and fluoroscopy times exceeding 30 min (Figs. 3 and 4). In the first case, a large hiatal hernia was identified as an anatomical abnormality (documented in the endoscopic report). In the other case within this age group, no anatomical anomalies were noted. Another notable case involved a 15-year-old female patient (age group 4) with successful tube placement, yet associated with a DAP of 198 cGy·cm^2^ and a fluoroscopy time of 27 min. The procedure report described repeated retrograde deflection of the tube tip at the level of the ligament of Treitz, which complicated final fully successful positioning. This case is a distinct outlier as shown in Fig. 3.

In the subgroup of cases with fluoroscopy times exceeding 15 min (*n*=15), the median DAP was markedly elevated at 34.0 cGy·cm^2^ (IQR, 52.7 cGy·cm^2^; range, 1–343 cGy·cm^2^). This subgroup included the above-mentioned patient with the highest recorded dose in the cohort (343 cGy·cm^2^). In this subgroup, nine cases were fully successful, one achieved partial success with duodenal placement due to malrotation, and five were unsuccessful; among the latter, three had no documented cause, one involved tube kinking at the ligament of Treitz with subsequent displacement into the stomach (Fig. 2), and one occurred in a patient with a hooked stomach in superior mesenteric artery syndrome.

Three patients underwent a high number of procedures. In a patient with 17 procedures (aged 6–14 years), fluoroscopy times were mostly below 15 min, with one procedure lasting approximately 22 min; three procedures were unsuccessful and required endoscopic placement. The patient with 13 procedures (aged 8–17 years) had exclusively successful placements, all with fluoroscopy times below 10 min. In the patient with nine procedures (aged 3–7 years), fluoroscopy times were generally below 5 min; one procedure was unsuccessful and one scheduled exchange required 13 min.

There were no statistically significant examiner-related differences in fluoroscopy time or DAP.

## Discussion

This study presents one of the largest pediatric cohorts to date, investigating the safety, technical success, and influencing anatomical and procedural factors of fluoroscopically guided PEG-J placement in children. Our results demonstrate a high technical success rate and confirm the procedure’s feasibility without sedation across all pediatric age groups, including infants and adolescents. In our cohort, procedural success and fluoroscopy time were primarily influenced by anatomical factors. Our findings emphasize the importance of careful fluoroscopic assessment of anatomy, early recognition of technical limitations, and individualized decision-making regarding continuation or termination of the procedure. In clinical practice, absence of incremental technical progress despite optimization maneuvers - especially in the setting of prolonged fluoroscopy time (approximately 15 min or more) - may serve as a pragmatic indicator to reconsider continuation of the procedure.

One of the largest published series of fluoroscopically guided PEG-J placements comes from Uflacker et al., who reported 391 adult cases with technical success rates of 91.9% for initial placements and 94.2% for replacements [11]. In comparison, success rates in our pediatric cohort were slightly lower for first-time insertions (81%) and for replacements (87%; Table 3), likely reflecting pediatric-specific anatomical and size-related challenges, especially in infants and young children. Uflacker et al. observed significantly longer fluoroscopy times in failed procedures. Notably, similarly, in our cohort, prolonged fluoroscopy - defined as >15 min - was associated with technical failure (Fig. 4). While Uflacker et al. did not report radiation dose metrics, our findings underscore the importance of monitoring both procedural time and radiation exposure, particularly in children where dose sensitivity is higher.

Karabulut et al. reported on a small series of five children with failed gastric feeding due to reflux or anatomical challenges in whom fluoroscopic jejunal placement improved nutritional outcomes (weight gain) [13]. None of the patients required anesthesia, and no surgical interventions were necessary - highlighting the minimal invasiveness and feasibility of the technique. The clinical indications and procedural setting closely mirrored those of our cohort. While the sample size was limited, our series substantially expands this evidence base, emphasizing that this method is broadly applicable in neurologically impaired or surgically pre-treated children with feeding intolerance.

Endoscopic jejunal tube placement using ultra-thin or mini-endoscopes and one-step percutaneous gastrojejunostomy under image guidance represent established alternatives to fluoroscopic conversion of an existing gastrostomy [16–18]. Although these approaches may reduce radiation exposure, they typically require sedation or general anesthesia and specialized expertise, particularly in younger or neurologically impaired children. In contrast, fluoroscopic placement via a mature gastrostomy tract can be performed without sedation and provides precise real-time visualization, which is particularly advantageous in chronically ill pediatric patients who frequently already have a gastrostomy with a non-balloon device at the time jejunal feeding becomes necessary. Supporting evidence from adult literature includes a comparative study by Kim et al., in which primary fluoroscopic gastrojejunostomy placement required sedation but demonstrated a low failure or malposition rate of 7.4% [12]; although procedural conditions differ from our pediatric cohort, these findings are consistent with our experience and underscore the technical reliability of fluoroscopic approaches.

From a broader perspective, Deliwala et al. conducted a systematic review and meta-analysis comparing PEG-J and direct percutaneous endoscopic jejunostomy, predominantly in adult populations [10]. They reported pooled technical success rates of 94% for PEG-J and 87% for direct jejunostomy, which is similar to our results. However, this study also included post-interventional tube dysfunction and clinical course, which were not within the scope of our analysis. More importantly, the fact that most available data are based on adult patients highlights the scarcity of pediatric-specific evidence, particularly for age-stratified technical outcomes and anatomical anomalies.

A study from 1994 reported a success rate of 97% for fluoroscopically guided nasojejunal tube placement with a median fluoroscopy time of 5.5 min - closely matching our fluoroscopy duration (median, ~5 min) [19]. The authors observed prolonged procedure times and increased difficulty in cases with anatomical variants, such as malrotation - paralleling our own findings, where aberrant intestinal anatomy was a leading cause of technical failure and extended fluoroscopy times. These similarities reinforce the generalizability of our observations and highlight the relevance of individual anatomical factors when planning and executing jejunal tube placement in children (Fig. 2).

Vitta et al. reported age-stratified dose values for nasojejunal tube placement in children, with mean DAP values ranging from 2.3 cGy·cm^2^ (age, <1 year) to 13.3 cGy·cm^2^ (>15 years) [20]. Although overall DAP values in our cohort were higher - except for a comparable median in age group 4 - the median doses of successful-only placements were slightly lower than the benchmark values in age groups 4 (6.8 cGy·cm^2^ vs. 7.8 cGy·cm^2^) and 5 (6.6 cGy·cm^2^ vs. 9.4 cGy·cm^2^; Fig. 3). These findings suggest that radiation dose levels differ between nasojejunal and gastrojejunal tube placements, likely due to procedural complexity and anatomical variability. To date, our study represents one of the largest pediatric cohorts with age-stratified dose data for gastrojejunal tube placement, providing important reference in a field where such data are still scarce.

The following limitations should be acknowledged: The retrospective design introduces potential biases such as incomplete documentation of procedural difficulties or anatomical variations. Given the single-center nature of the study, results may not be generalizable to institutions with different equipment or levels of expertise. We recorded fluoroscopy time and DAP but lacked long-term follow-up data on nutritional outcomes or tube-related complications. While our sample is the largest pediatric PEG-J cohort reported, it remains small relative to adult studies and may lack power to fully explore subgroup differences. Although procedural tolerance was not formally assessed, tube placement was generally well tolerated in our clinical experience, with procedural discomfort mainly related to local gastrostomy site irritation or positioning rather than the intervention itself.

In conclusion, fluoroscopically guided PEG-J placement is a safe and effective technique for post-pyloric enteral access in children, including infants and adolescents. It avoids general anesthesia or sedation, offers high technical success, and maintains low complication rates, making it a valuable alternative for pediatric patients with complex gastrointestinal or neurological conditions.

## Data Availability

The data that support the findings of this study are not openly available due to reasons of sensitivity and are available from the corresponding author upon reasonable request.
